# Oral rehabilitation with dental implants in patients with systemic sclerosis: A systematic review

**DOI:** 10.1002/iid3.812

**Published:** 2023-03-17

**Authors:** Seyed Ali Mosaddad, Reza Abdollahi Namanloo, Raziye Ghodsi, Yasaman Salimi, Masumeh Taghva, Meshkat Naeimi Darestani

**Affiliations:** ^1^ Student Research Committee, School of Dentistry Shiraz University of Medical Sciences Shiraz Iran; ^2^ Department of Dentistry Bogomolets National Medical University Kyiv Ukraine; ^3^ Department of Periodontics, Dental School Shiraz University of Medical Sciences Shiraz Iran; ^4^ Student Research Committee Kermanshah University of Medical Sciences Kermanshah Iran; ^5^ Department of Prosthodontics, School of Dentistry Shiraz University of Medical Sciences Shiraz Iran; ^6^ Department of Periodontics, Dental Faculty Semnan University of Medical Sciences Tehran Iran

**Keywords:** dental implants, implant‐supported dental prosthesis, survival rate, systemic scleroderma, systemic sclerosis

## Abstract

**Objective:**

To assess the influence of systemic sclerosis (SSc) on the survival rate of dental implants in SSc patients receiving implant‐supported treatments.

**Methods:**

The Preferred Reporting Items for Systematic Reviews and Meta‐analysis (PRISMA) Statement and the Cochrane Collaboration's guiding principles were followed during the study's execution. The data from three databases, PubMed, Google Scholar, and Scopus, available until January 2023, were used to compile the material for our research. Only English‐language publications were submitted for this research and evaluated based on their titles, abstracts, and full texts. For performing a quality assessment, quality scores were calculated.

**Results:**

The total number of patients and implants studied were 37 and 153, respectively, all having had scleroderma. The patients’ ages ranged from 28 to 77 years old, with a mean (*SD*) age of 58.16 (12.88). All the patients in the case reports and most in the case series study were female. The range of follow‐up duration was from 1 to 10 years. In case report studies, the survival rate was 100%; in case series, it was 89.2%.

**Conclusion:**

The SSc status had no discernible impact on the implant survival rate. Implant‐based treatments in SSc patients should not worsen the overall morbidity and should not conflict with systemic treatments. Before starting implant therapy, a thorough risk assessment is essential, though.

## Graphical abstract

In this systematic review, we aimed to determine if systemic sclerosis as an autoimmune condition could impact the treatment outcomes and success rate of dental implants. Our study found that the functional advantages of dental implants and improved quality of life can generally outweigh the risks, making systemic disease control more critical than the disordered nature.

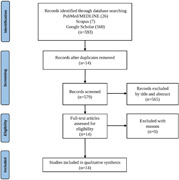

## 1 INTRODUCTION

As an autoimmune disorder, scleroderma or systemic sclerosis (SSc) affects blood vessels and connective tissues^1^ through pathophysiological processes such as obstructive vasculopathy of small vessels and immunological deregulation followed by fibroblast inflammation and dysfunction, which increases extracellular matrix deposition.^2^ The heart, lungs, kidneys, and gastrointestinal system can all be affected by this condition due to mucosal and skin fibrosis.^3^ Two main clinical subgroups, limited systemic scleroderma (lSSc) and diffuse systemic scleroderma (dSSc), can be distinguished. Differentiation is possible based on the disease's progression, the implication of the internal organs, and the particular antibodies involved.^4^ Calcinosis cutis, Raynaud's phenomenon, esophageal dysmotility, sclerodactyly, and telangiectasia are a few of the distinctive signs of lSSc.^5^ Skin stiffness and itching are early signs of dSSc.^6^ Occasionally, individuals will exhibit musculoskeletal pain that resembles an inflammatory joint disease. Exercise‐induced dyspnea and weight loss are common symptoms. Weight loss is typically accompanied by decreased food intake or appetite.^7^ Early‐stage dSSc patients may have lower‐limb edema, muscle weakening, or tiredness.^8^

Like many other rheumatological diseases, this condition affects females more frequently than males. Any age, including childhood, can experience SSc manifestations. According to reports, the highest onset age fell between 20 and 50. However, the research found it was later, between 55 and 69 years.^9^ Reports have linked later disease onset, male gender, and African‐Caribbean ancestry to a more aggressive disease trajectory.^10^ At this moment, there is only an incomplete understanding of the causes and pathophysiology of SSc. A set of multiple factors is postulated to have a role. It is perceived that patients who develop SSc have a cardinal genetic predisposition and then are exposed to an environmental factor that instigates the inflammatory and fibrotic cascades.^11^

The mouth and face's cutaneous, mucosal, and muscular compartments are frequently engrossed in SSc, with lips being the most commonly affected site.^12^ There are sporadic manifestations of regional sensory signs, such as glossopharyngeal or trigeminal neuralgia/neuropathy. One of the earliest signs of SSc is the scleroglosson or frenulum of the tongue shortening and thickening.^3^ The characteristic radial folds at the corners of the mouth (tobacco pouch mouth) and a progressive constraint in mouth opening (microstomia) due to an overabundance of collagen being deposited in the skin and perioral tissues are the outcomes of the orbicularis oris muscle involvement.^13^ Consequently, patients often complain about aesthetic concerns with skin sclerosis and telangiectasia, reduced ability to open their mouth, altered dentition, dental surgery difficulties, or dry mouth. Because of salivary gland fibrosis, inadequate salivary flow (xerostomia) compromises the oral cavity's ability to buffer the acid produced by bacterial activity and gastric juice (caused by acid reflux), which erodes teeth and leads to dental caries and periodontal diseases.^14^ As a result, wearing mucosal‐borne dentures, oral care, and dental procedures are all compromised.^15^

Peri‐implantitis and periodontitis refer to the progressive inflammation of the supporting structure of implants and teeth. An imbalance between the internal immunological response of the host and the foreign body response causes these conditions,^16^ which in turn leads to implant surface contamination, alveolar bone loss, tooth/implant loss, and tooth mobility.^17^ The distinctive clinical profile in patients with periodontal inflammation and SSc includes widened periodontal ligament,^18^ decreased bleeding index and probing depth, and increased periodontal attachment loss.^2^ Patients with SSc have been found to have periodontal microcirculation disorders, idiopathic tooth and bone resorption, and pathologic fractures in the mandible.^19^ SSc patients need an adequately planned therapeutic approach to maintain or recover chewing function due to the complexity of extraoral and intraoral symptoms. The patients’ requirement for multiple smaller meals and vast amounts of hydration throughout the day is one determinant in this regard.^12^ Due to potential disease‐related motility disorders that impact the esophagus, food should be chewed properly to prevent choking and ensure adequate nutritional digestion.

The diagnosis, treatment planning, and managing patients with SSc are all directly affected by the presence of xerostomia, microstomia, reduced vascularity (compromising tissue healing), tissue ischemia due to continuously increased oral tissue pressure brought on by fibrosis, bone resorption, and tooth mobility. Microstomia, finger deformities, and/or restricted manual skills cause daily oral hygiene activities and the wearing and removal of conventional removable dentures to be challenging for patients as the disease progresses.^3^ Hyposalivation interferes with the prosthetic's efficient retentive properties and increases the chance of denture‐associated mucosal ulcerations. Additionally, even though SSc patients may endure the initial treatment plan, as the condition advances, continued bone loss in edentulous jaws may make it difficult to maintain the current restoration, and adjustments or replacement options might be needed.^20^

Dental implants have been utilized as artificial tooth roots to replace missing teeth by supporting prosthetic superstructures from single crowns to fixed and removable prostheses.^21^ It is commonly known that dental implants offer many advantages, such as reducing bone resorption and oral mucosal ulcers brought on by unfit prosthetics and boosting prosthetic retentive features.^22^ As a result, implant‐retained prostheses have improved as a potential rehabilitating option for SSc patients who are partially or completely edentulous. Since in SSc, systemic and periodontal inflammation share pathogenic pathways^23^ and the long‐term success rate of dental implants is affected by peri‐implant inflammation; therefore, it is uncertain if these patients would benefit from dental implants and implant‐supported prostheses to avoid comprehensive tangency between prostheses and sensitive oral mucosa. However, limited research has been done on this type of rehabilitation for SSc patients.^20^ Therefore, this study aimed to assess the survival rate of dental implants in SSc patients.

## 2 MATERIALS AND METHODS

In this study, the influence of SSc on dental implants was explored. The search strategy, screening, and data extraction were all checklist‐based. The Preferred Reporting Items for Systematic Reviews and Meta‐analysis (PRISMA) were also adhered to.^24^ The protocol for this systematic review was registered and is visible in Open Science Framework (https://doi.org/10.17605/OSF.IO/4PF95).

### 2.1 Search strategy

A comprehensive search was conducted on January 2023 in Pubmed, Scopus, and Google Scholar independently by two authors (S.A.M and Y.S). The search strategy is summarized in Table 1. Initially, studies were searched and retrieved. The papers were vetted based on their title and abstract after duplicates were discarded. Finally, studies that met the inclusion criteria were included. Cohen's Kappa coefficient was used to calculate the inter‐rater reliability between the evaluators for the literature screening procedure. The kappa value (к) was determined based on the frequencies of precise agreements between reviewers.

**Table 1:** Search strategies for PubMed and Scopus databases.

Database	Search strategy	Additional filters
PubMed/MEDLINE	(“Dental Implants”[Mesh] OR Implant, Dental OR Implants, Dental OR Dental Implant OR Dental Implants, Mini OR Dental Implant, Mini OR Mini Dental Implant OR Mini Dental Implants OR Dental Prostheses, Surgical OR Dental Prosthesis, Surgical OR Surgical Dental Prostheses OR Surgical Dental Prosthesis OR Prostheses, Surgical Dental OR Prosthesis, Surgical Dental) AND (Systemic Sclerosis OR Sclerosis, Systemic OR Systemic Scleroderma OR Scleroderma, Progressive OR Progressive Scleroderma OR Diffuse Cutaneous Systemic Sclerosis OR Sudden Onset Scleroderma OR Scleroderma, Sudden Onset OR Sclerodermas, Sudden Onset OR Sudden Onset Sclerodermas OR Diffuse Systemic Sclerosis OR Diffuse Systemic Scleroses OR Scleroses, Diffuse Systemic OR Sclerosis, Diffuse Systemic OR Systemic Scleroses, Diffuse OR Systemic Sclerosis, Diffuse OR Diffuse Scleroderma OR Sclerosis, Progressive Systemic OR Progressive Systemic Sclerosis OR Systemic Sclerosis, Progressive OR Limited Scleroderma OR Limited Systemic Scleroderma OR Limited Systemic Sclerodermas OR Scleroderma, Limited Systemic OR Sclerodermas, Limited Systemic OR Systemic Scleroderma, Limited OR Systemic Sclerodermas, Limited OR “Scleroderma, Systemic”[Mesh])	English, January 4^th,^ 2023
Scopus	TITLE‐ABS‐KEY (dent* AND implant* AND (systemic AND sclerosis* OR diffuse AND scleroderma* OR limited AND scleroderma* OR systemic AND scleroderma* OR crest AND syndrome OR diffuse AND sclerosis OR limited AND sclerosis))	English, January 4^th,^ 2023

### 2.2 Inclusion and exclusion criteria

Case reports/series studies published within the last 30 years that investigated the role of systemic scleroderma on the survival of dental implants and its outcomes were included. The exclusion criteria were non‐English language, reviews, posters, abstracts, and studies with insufficient data.

### 2.3 Quality assessment and data extraction

The studies' quality was evaluated using the following guidelines (https://jbi.global/critical-appraisal-tools). All included studies underwent a quality assessment independently by two reviewers (Y.S and R.A.N). Then, data extraction was conducted by two reviewers (S.A.M and R.G), and the required information was extracted and recorded using a standardized Excel datasheet. The extraction form contained the last name of the first authors, published year, country of origin, type of the study, study setting (age, sex, and follow‐up duration), sample size, the survival rate of implants, and outcome. In disagreements between reviewers, a third author (M.T) was consulted.

## 3 RESULTS

### 3.1 Study selection

**Figure 1:**
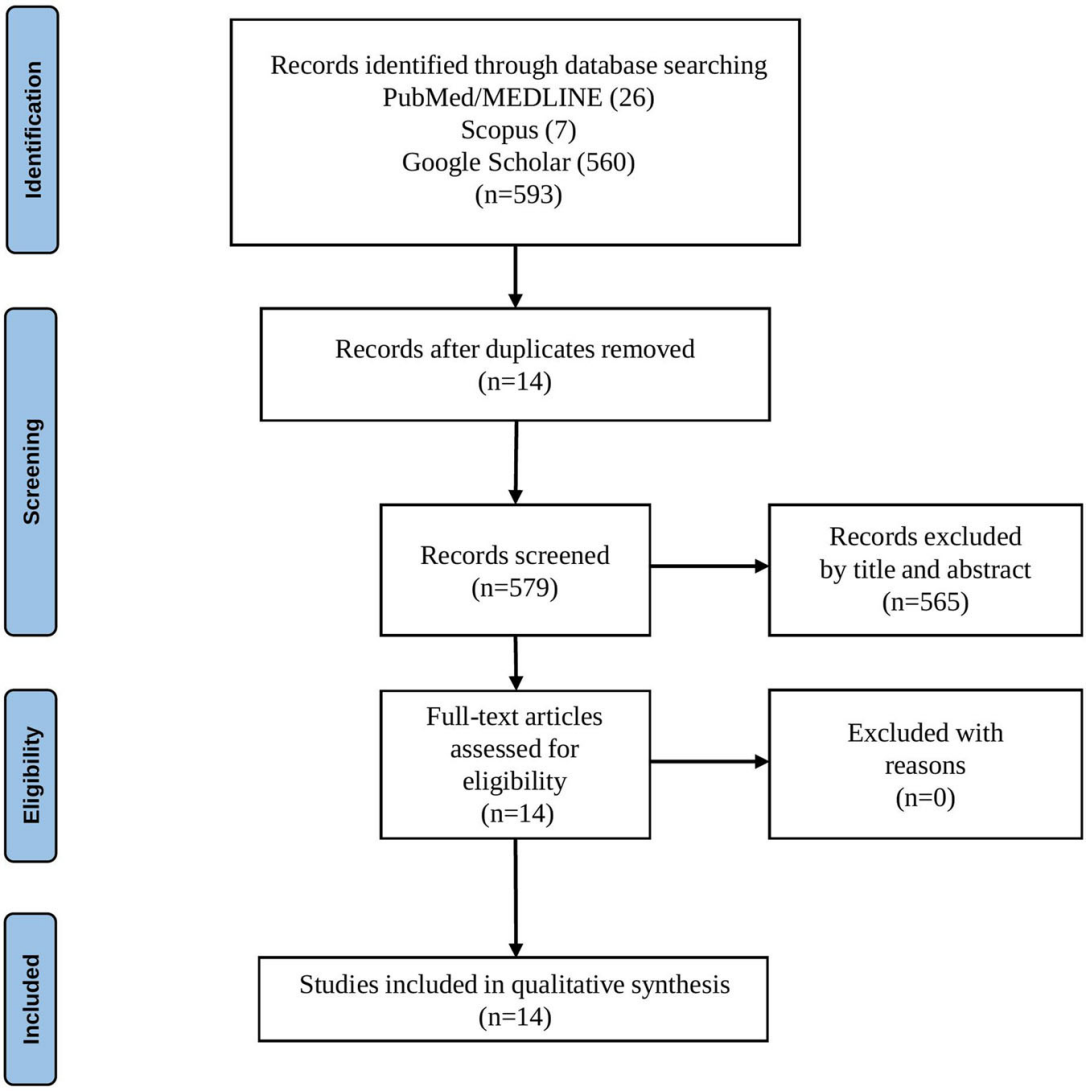
PRISMA flowchart illustrating the procedures and results of paper screening and selection for this systematic review. PRISMA, Preferred Reporting Items for Systematic Reviews and Meta‐analysis.

Initially, 593 articles were found. Duplicates were removed and based on the title and abstract, 579 remaining studies were reviewed. After analyzing the full text of remained articles, 565 records were excluded due to being irrelevant. In this systematic review, 14 papers were included (Figure 1). Cohen's Kappa coefficient revealed almost perfect agreement between the inter‐raters (к = 0.86).

### 3.2 Study characteristics

This study reviewed 14 investigations, of which one study was a case series,^3^ and 13 remaining articles were case reports (Table 2). The total number of patients studied in included investigations was 37, all dealing with scleroderma. Overall, 153 implants were studied in the included experiments. Three studies were carried out in the United States,^19^, ^25^, ^26^ two in Israel,^20^, ^27^ and other studies were performed in Switzerland, Croatia, Canada, Brazil, Argentina, Turkey, the United Kingdom, India, and Germany.^1^, ^3^, ^28^, ^29^, ^30^, ^31^, ^32^, ^33^, ^34^

**Table 2:** Characteristics of the included studies.

Author	Year	Country	Number of patients	Number of implants	Type of edentulism	Type of the prosthesis	Type of study	Age	Sex	Follow‐up duration	Survival rate	Outcome(s)
Jackowski et al.^3^	2021	Germany	24	72 Including: 56 implants (Straumann, Basel, Switzerland) 15 implants (Thommen Medical SPI, Grenchen, Switzerland) One implant (Friadent, York, Pennsylvania, USA)	Single tooth missing Partial Edentulism Complete Edentulism	Variable	Retrospective case series	59.6 ± 13.08 years (mean ± *SD*)	22 Female 2 male	Average 5.9 years (range: 0.7–17.8)	89.2% (95% CI: 79.1–95.6)	The mean value of peri‐implant pocket depth: 2.4 and 2.8 mm The mean value of interincisal distance: 29.54 mm A positive relation between sulcus bleeding index and plaque index and a positive association between periimplant pocket depth and plaque index. An implant can be inserted and maintained successfully in these patients.
Garcés Villalá and Zorrilla Albert^1^	2021	Argentina	1	12 Implants (Odontit® implant, Argentina)	Complete Mandibular and Maxillary Edentulism	Fixed and self‐cleaning screw‐retained hybrid prosthesis	Case report	54 years old	Female	10 years	100%	Bone loss after 10 years: 1.26 mm for the maxilla 1.17 for the mandible. Showed similar bone loss compared to healthy patients. The function was recovered completely.
Langer et al.^27^	1992	Israel	1	Two I.M.Z. implants (Interpore International, Irvine, California, USA)	Complete Maxillary Edentulism	A Reinforced Maxillary Complete Overdenture	Case report	54 years old	Female	–	100%	Dental implants, osseointegrated and maintained functionally stable.
Raviv et al.^28^	1996	Canada	1	Three implants (Nobel Biocare, Göteborg, Sweden)	Complete Mandibular Edentulism	Mandibular implant‐retained overdenture	Case report	65 years old	Female	–	100%	Dental implants osseointegrated and maintained functionally stable.
Patel et al.^29^	1998	United Kingdom	1	Four implants (Nobel Biocare, Göteborg, Sweden)	Partial Maxillary and Mandibular Edentulism	Mandibular implant‐supported FPD	Case report	54 years old	Female	–	100%	Dental implants osseointegrated and maintained functionally stable.
Öczakir et al.^30^	2005	Switzerland	1	Eight ITI implants (Straumann, Basel, Switzerland) (four maxillary and four mandibular)	Complete Maxillary Edentulism Partial Mandibular Edentulism	Maxillary: Implant‐retained overdenture with a horseshoe design and extended bar support. Mandibular: FPD	Case report	64 years old	Female	5 years	100%	Prostheses and implant survival were 100%. All implants demonstrated stable function.
Haas^25^	2002	USA	1	Seven implants (Nobel Biocare, Göteborg, Sweden)	Complete Maxillary Edentulism Partial Mandibular Edentulism	Maxillary Implant‐retained FPD	Case report	49 years old	Female	–	100%	Dental implants osseointegrated and maintained functionally stable.
Zigdon et al.^20^	2011	Israel	1	12 MIS implants (M.I.S. Implants Technology, Israel) (six maxillary and six mandibular)	Complete Mandibular and Maxillary Edentulism	Maxillary and Mandibular fixed‐detachable implant‐supported restorations	Case report	45 years old	Female	3 years	100%	No radiological evidence of peri‐implant bone loss Good oral hygiene and clinical condition were evident.
Nam et al.^26^	2012	USA	1	14 NobelReplace™ implants (Nobel Biocare, Göteborg, Sweden) (eight maxillary and six mandibular)	Complete Mandibular and Maxillary Edentulism	Implant‐supported maxillary and mandibular FPDs	Case report	71 years old	Female	–	100%	Full mouth reconstruction is probable in patients with scleroderma.
Zanettini et al.^31^	2017	Brazil	1	Three SLActive Standard Plus implants (Straumann, Basel, Switzerland)	Partial Maxillary Edentulism	Implant‐supported Maxillary FPD	Case report	65 years old	Female	2 years	100%	At follow‐ups, tissue stability was good, and there were no pocket formation and radiographic abnormalities
Smojver et al.^32^	2021	Croatia	1	Two BLT SLActive Roxolid RC implants (Straumann, Basel, Switzerland)	Partial Maxillary Edentulism	Implant‐retained Maxillary FPD	Case report	55 years old	Female	1 year	100%	Rehabilitation with implants was successful, and no pain or failure was experienced.
Mehta et al.^33^	2021	India	1	Two Touareg™ implants (Adin Dental Implant Systems Ltd., India)	Complete Mandibular Edentulism	Mandibular Implant‐supported Collapsible Overdenture	Case report	28 years old	Female	–	100%	Prosthodontic management, which was supported by the implant, was successful
Bayram et al.^34^	2022	Turkey	1	Seven implants (Straumann, Basel, Switzerland)	Partial Maxillary and Mandibular Edentulism	Maxillary and Mandibular Implant‐assisted fixed prostheses (screw‐retained)	Case report	57 years old	Female	4.8 years	100%	After 4.8 years, there were no discernible signs of peri‐implant disease, including inflammation and bleeding. On radiography, there was no evidence of crestal bone loss surrounding the implants.
Baptist ^19^	2016	USA	1	Five implants (Implant Direct Sybron, CalabasasHills, California, USA)	Partial Maxillary Edentulism	Maxillary Implant‐supported fixed prostheses	Case report	61 years old	Female	2 years	100%	Improved mastication. No discernible crestal bone loss on radiographs. No visible indicators of peri‐implant disease, like bleeding or inflammation, were found.

Abbreviation: FPD, fixed partial denture.

### 3.3 Patients characteristics and implant survival rate

The patients’ ages varied from 28 to 77; the mean (*SD*) age of patients studied was 58.16 (12.88) years old, of which 94.6% were female. All the patients in the case reports were female, while in the case series study,^3^ the population group involved 24 female and two male patients (5.4%). The follow‐up duration range was from 1 to 10 years. The survival rate in all case reports was 100%; the survival rate in the case series was 89.2%. Table 2 presents data on the characteristics and outcomes extracted from the included papers.

### 3.4 Quality assessment

**Figure 2:**
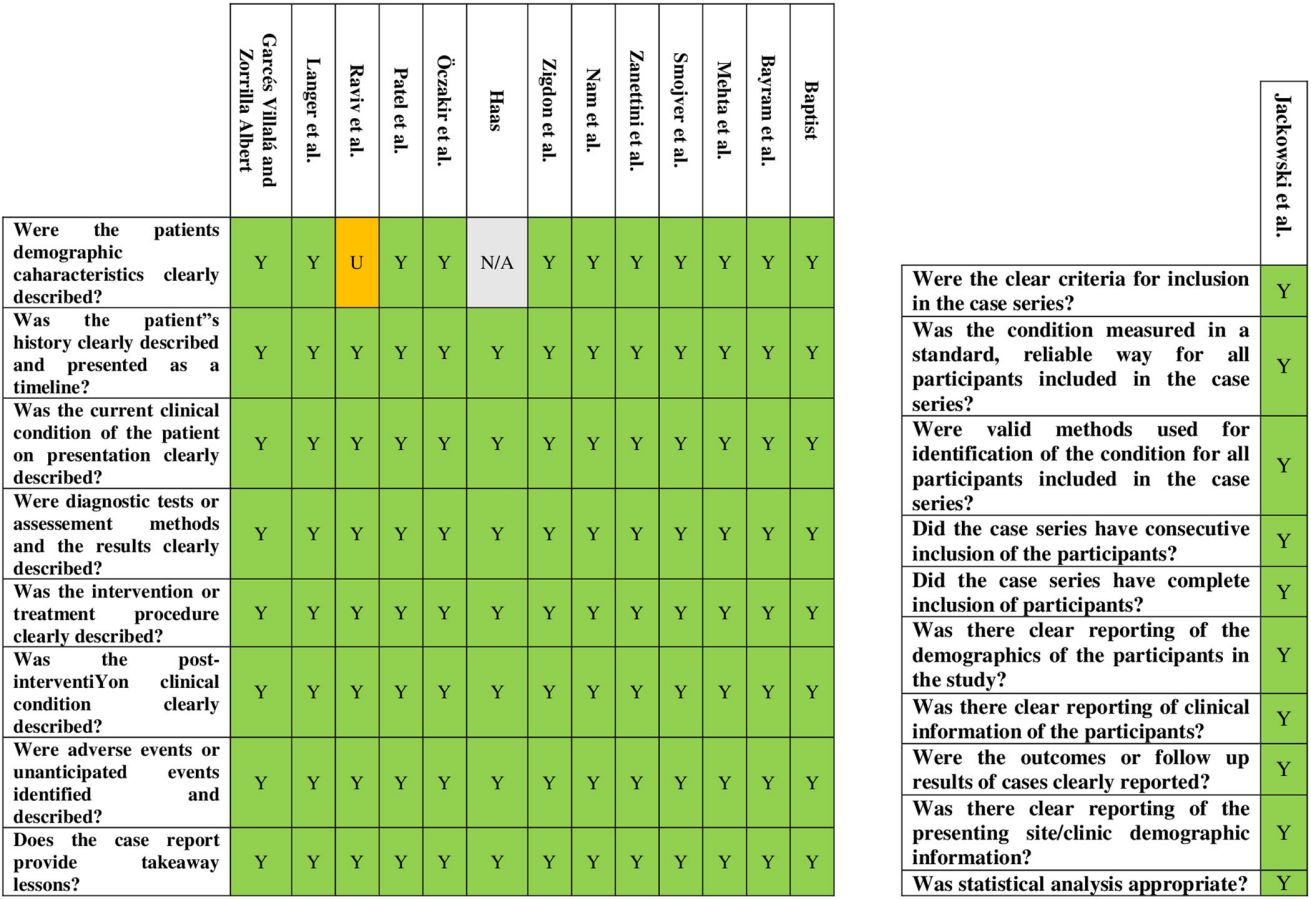
Results of the quality assessment for included studies. N/A, not applicable; Y, yes; U, unclear.

The findings of the quality assessment of the included publications are illustrated in Figure 2. Based on the checklist used to score papers, all studies achieved complete scores, except for two studies^25^, ^28^ in which the demographic characteristics were unclear or not applicable.

## 4 DISCUSSION

This systematic review study reviewed 14 case reports/series discussing the current evidence on the role of scleroderma in the survival rate of dental implant treatment in the afflicted population. The implant survival rate was not significantly affected by SSc. Dental implants’ therapeutic potential for individuals with SSc is now only understood relying on clinical findings reported in single case reports and limited case series. An implant's clinical results and survival rate determine the treatment's success rate. Choosing dental implants as a treatment option in SSc patients may be influenced by oral implications, such as hyposalivation, progressive microstomia, disturbed oral vascularity, tooth mobility, and decreased bone turnover, even though our data showed encouraging results in this area.

Additionally, dental clinicians should consider the disease's progressive character when designing a long‐term care strategy. Furthermore, it is crucial to account for any patient‐specific conditions, such as those brought on by the disease and medication (often bisphosphonates, anticoagulants, and corticosteroids), all of which are leading risk factors that should be identified before dental implants are placed.^35^ Furthermore, the accessibility, high caries risk, and bone quality provide an extra level of possible complications that should not be understated. Regarding implant therapy in SSc patients, Jackowski et al.^3^ reported that patients could benefit from dental implants to substitute single areas of tooth missings or to support removable dentures with a high success rate. Raviv et al.^28^ also suggested that an implant‐retained overdenture could be a successful prosthetic option for edentulous patients having chronic progressive systemic diseases like SSc. However, in SSc, fibrosis of the perioral dermis causes the corners of the mouth to become less elastic, which may make it more challenging to get access during surgery and restrict the use of any form of removable prostheses.^36^ To overcome these limitations, multiple approaches exist. For example, placing implant drills axially might not be possible; thus, short implants or angled abutments might be regarded as alternates.

Additionally, surgically broadening the mouth corner (commissurotomy) may be possible, mainly if the mouth opening is less than 16 cm.^37^ However, impaired postsurgical wound healing can lead to scarring, which can cause microstomia to return. Using foldable or collapsible prostheses with easy retrieval is a therapeutic alternative to commissurotomy in prosthesis construction. These prostheses are fabricated of pliable silicone and equipped with hinges or attachments that make them integrated to offer rigidity. The patient's ability to properly wear and remove the prosthetic denture is equally as crucial as the prosthetic denture's design. Therefore, as long as the patient has no physical disabilities, these prosthetic choices function. According to a clinical report by Mehta et al.,^33^ an SSc patient with microstomia and an atrophic mandibular ridge was given an implant‐supported foldable overdenture with an O‐ring attachment. They concluded that flexible or foldable dentures could promote fitness and simplicity of usage, and implant‐supported overdentures may be considered when dealing with severely atrophic ridges by altering prosthetic processes from impression to delivery. Furthermore, removable dentures frequently need to be adjusted in size to maintain functionality due to the perioral fibrosis process, which commonly develops quickly.

On the other hand, selecting fixed‐type restorations for patients has been reported to have superior results compared to removable dentures. To provide surgical/prosthetic accessibility, this treatment modality is indicated when the inter‐incisal distance has not yet been reduced to 3 cm. In this regard, Finding an appropriate prosthesis for a patient with SSc presented significant challenges, according to Patel et al.^29^ Due to the inadequacy of the denture‐bearing area, altered peripheral seal, and limited teeth to provide support and retention, a conventional removable partial denture was not viable. These difficulties were resolved via an implant‐supported fixed partial denture (FPD). Bayram et al.^34^ designed implant‐retained fixed restorations for their patient. According to the patient, this treatment was crucial to how she perceived her SSc and mastication's ability to recover. The intraoral examination after 4.8 years revealed no evidence of peri‐implant inflammation, bleeding, or other discernible symptoms of peri‐implant pathology. They suggested implant‐assisted fixed restorations in SSc patients would be a promising therapeutic choice. Therefore, everyday prosthesis removal would be challenging due to the progressive microstomia in patients with physical impairments. Beyond the initial improvement in function and mastication observed in all treated edentulous/partially edentulous patients, a fixed implant prosthesis has inherent advantages for patients with SSc. The possibility of fabricating fixed implant‐retained restorations leads to a smaller prosthesis, making clinical usage easier. The advantage of retrieving, repairing, and modifying the prosthesis is better maximized when progressive microstomia or more tooth/implant loss leads to prosthetic complications in a patient with a segmented, screw‐retained prosthesis. A fixed prosthesis will be maintainable with at‐home oral hygiene products and function despite a patient's loss of manual dexterity over the progression of SSc. This opposes removable dentures, which might hinder at‐home oral care and increase implant complications due to infrequent removal following diminished motor function, accelerated microstomia, gingival fibrosis, and ongoing alveolar bone resorption.^35^ For partially and completely edentulous patients with lSSc who could not use conventional dentures because of their anatomical and functional limitations, Garcés Villalá and Zorrilla Albert^1^ offered a fixed rehabilitation using dental implants. Following 10 years of monitoring, the implants had experienced bone loss comparable to that seen in healthy patients, and no peri‐implant diseases were found. Function, aesthetics, and self‐esteem were reestablished by this therapy.

Hass detailed how a patient with SSc was treated in considering FPDs. It was shown that with little prosthetic creativity, it is feasible to construct long‐span implant‐supported FPDs for these individuals.^25^ Nam et al.^26^ also presented a successful transition from natural teeth to implant‐supported FPDs for an SSc patient. In this case, the progressive restriction of the mouth opening was a further problem. Because of her restricted bilateral extension of the perioral musculature, the authors had trouble getting the impression trays inside her mouth to take impressions of her temporary restorations. To resolve the problem, the authors had to transfer her provisional restorations to the master cast to create provisional restoration casts. Another approach to overcoming impression‐taking limitations is sectional impression methods for edentulous patients.

Research suggests significant correlations between SSc and periodontal inflammation.^2^, ^38^, ^39^, ^40^ Numerous factors shared between SSc and periodontal disorders, including vascular dysfunction, increase the risk of inflammation and decrease the success rate of implant‐based treatment in patients with SSc and periodontitis.^41^ In addition, bone turnover is affected in SSc,^40^, ^42^ which as a hypothesis, might accelerate peri‐implant bone loss when inflammation is present, affecting the stability of the peri‐implant marginal bone and, consequently, the long‐term success of the treatment.^43^ Hypoxia is critical in the progression of SSc and local bone dysregulation. In a hypoxic environment, osteocytes apoptosis and osteoclasts proliferation and differentiation can be promoted through stimulation of the receptor activator of nuclear factor‐κB (RANK) and RANK ligand (RANKL) pathways by elevated hypoxia‐inducible factor 1‐alpha, advanced glycation end products (AGEs), interleukin 6, vascular endothelial growth factors and the receptor for AGEs. Osteoblasts’ activities can also be endorsed through enhancement in Wnt signaling, which can cause diverse bone affections as typically observed in the disease.^44^ These factors are the biomarkers of peri‐implant inflammatory conditions.^45^, ^46^ Therefore, a high level of patient motivation, the foundation for their willingness to undergo periodic intraoral follow‐up evaluations, is another requirement for long‐term implant survival. Frontal linear scleroderma “*en coup de sabre*” in a female patient was described by Zanettini et al.^31^ Their findings suggest that oral reconstruction could be accomplished. Still, careful consideration of the surgical strategy, risks, and advantages is primarily required. They mentioned that avoiding serious local complications requires early detection and diligent monitoring. Whether the implant‐retained prosthesis is meant to be fixed or removable, the patient must be able to maintain its longevity with proper at‐home dental care independently. To identify any pathological changes, routine checkups, twice yearly, should be conducted. As mentioned, the elevated inflammatory markers in patients with SSc necessitate improving oral care at the peri‐implant site for a successful outcome.^47^ Zigdon et al.^20^ reported good clinical results and oral hygiene following the reconstruction of the edentulous maxilla and mandible with dental implants in SSc patients who use systemic corticosteroids.

In general, it is advisable that an experienced surgeon handles the treatment of SSc patients and that an interdisciplinary approach by close collaboration among clinicians and rheumatologists/dermatologists is required for planning a comprehensive course of treatment and monitoring. Baptist et al.,^19^ in their case, considered a fixed‐implant supported rehabilitation due to the progressive microstomia, hyposalivation, chronic generalized periodontitis, reduced physical skill, and patient willingness. This case demonstrates the feasibility of successful implant recuperation for SSc patients with edentulous posterior maxilla following a detailed analysis of all limiting variables. It should be highlighted that the authors emphasize the necessity of successfully managing the scleroderma itself, which they believed to be a cause of the positive biologic outcomes in implant treatment.^48^ Additionally, the psychological advantages cannot be downplayed or overlooked. The patient might think that their premature tooth loss means they would eventually pass away from the disease. Control over the underlying health issues through one of the most obvious entry points for everyday activity, the teeth, is one of the most valued outcomes in this situation.

To the best of our knowledge, this systematic review is the first comprehensive study that has compiled data on the influence of systemic scleroderma on the clinical success and survival rates of dental implants in the affected community. However, the currently available investigations are primarily characterized by the absence of control groups and a low level of specificity and rely on a small but very heterogeneous patient collective. Systemic scleroderma and dental implant rehabilitation were not the subjects of any randomized controlled studies. Results were frequently summarized and not characterized as being patient‐specific, such as implant loss per patient. Since only English‐language studies were considered, some data may be missing. The urgent need for randomized controlled studies on systemic sclerosis and dental implants with larger patient populations is prompted by limited primary investigations.

## 5 CONCLUSION

There is scant evidence that the SSc disease has a negative impact on dental implants’ survival rates. As a result, it is difficult to draw a firm conclusion based on evidence about whether or not to consider dental implants in people with systemic sclerosis. Implant‐based approaches should not exacerbate the overall morbidity in SSc patients or restrict life‐saving interventions, risking the patient's health. Before implant therapy, comprehensive risk assessment using an interdisciplinary approach based on the patient's past and present medical history is crucial.

## AUTHOR CONTRIBUTIONS

*Conceptualization*: Yasaman Salimi and Seyed Ali Mosaddad. *Methodology*: Seyed Ali Mosaddad, Yasaman Salimi, and Reza Abdollahi Namanloo. *Software*: Seyed Ali Mosaddad. *Validation*: Seyed Ali Mosaddad, Masumeh Taghva, and Meshkat Naeimi Darestani. *Formal analysis*: Seyed Ali Mosaddad. *Investigation*: Yasaman Salimi and Seyed Ali Mosaddad. *Resources*: Yasaman Salimi and Seyed Ali Mosaddad. *Data curation*: Raziye Ghodsi and Seyed Ali Mosaddad. *Writing—original draft preparation*: Seyed Ali Mosaddad, Yasaman Salimi. *Writing—review and editing*: Masumeh Taghva and Seyed Ali Mosaddad. *Visualization*: Raziye Ghodsi and Reza Abdollahi Namanloo. *Supervision*: Masumeh Taghva. *Project administration*: Seyed Ali Mosaddad. All authors have read and approved the published version of the manuscript.

## CONFLICT OF INTEREST STATEMENT

The authors declare no conflicts of interest.

## Data Availability

Data used in structuring this study will be available upon request.
